# The Prevalence of Early Childhood Caries among 24 to 36 Months Old Children of Iran: Using the Novel ICDAS-II Method

**Published:** 2015-12

**Authors:** Hediyeh Toutouni, Mohammad-Reza Nokhostin, Bennett Tochukwu Amaechi, Abdol Hamid Zafarmand

**Affiliations:** a Dept. of Community Oral Health, Shahid Beheshti University of Medical Sciences School of Dentistry, Tehran, Iran.; b Dept. of Restorative Dentistry, Shahid Beheshti University of Medical Sciences School of Dentistry, Tehran, Iran.; c Dept. of Comprehensive Dentistry, Dental School, UT health science center, San Antonio, Texas, USA.

**Keywords:** ICDAS-II, Early childhood caries, Caries-free, Young children, Dental caries, Prevalence, Oral health, Iran

## Abstract

**Statement of the Problem:**

Early childhood caries is an important oral health issue. Finding its prevalence would predict the need for oral health promotion disciplines for specific age groups.

**Purpose:**

The aim of this study was to assess the caries experience of children living in Tehran, Iran. It also would evaluate the impact of gender, ethnicity, and socioeconomic status (SES) on this oral condition.

**Materials and Method:**

This epidemiological cross-sectional study was based upon stratified cluster random sampling. The samples consisted of 239 children (2- to 3- years old) registered in Tehran’s public healthcare centers for “Healthy Child Program”. Mothers of the recruited children were interviewed for the background data; then children were examined for the oral health status according to ICDAS-II (International Caries Detection and Assessment System) and WHO (World Health Organization) criteria. Statistical analyses were conducted using STATA.11 for SES classification considering six socioeconomic variables, and SPSS.21 for descriptive/analytical analyses.

**Results:**

Primary Component Analysis (PCA) demonstrated five classes of SES ranging from the lowest to the highest. The distribution of caries-free (CF) children was 10.87%, non-cavitated enamel caries (codes 01-02) were 28.03%, and about 61.1% had cavitated caries (codes 03-06). There was no significant difference in caries experience between the two genders. Cavitated lesions were more prevalent among Kurdish, who also had the least CF children. Caries prevalence, especially code 02, was more among children from 3^rd^ class SES (moderate level). Gender, ethnicity, or SES had no impact on the CF status of the children; however, ethnicity showed significant impact on the prevalence of extensive caries (codes 05-06).

**Conclusion:**

The result of the present study is indicative of high caries prevalence among 2 to 3 years old children residing in Tehran. It highlights the need for comprehensive oral health promotion disciplines for this age group.

## Introduction

Dental caries remains the highly prevalent oral lesion. It is a worldwide issue and continues to be one of the pandemics among children regardless of the socio-economic status and how well developed a country is.[1]Although caries is preventable, when developed it lives with the affected individual for life and constitutes economic and social burden. Once a restoration is placed, the tooth enters into a restorative cycle in which the several replacements would happen throughout the life.[2-8]

Iran’s National Oral Health Survey reported a prevalence of 47% dental caries among 3-year-old Iranian children in 1999 (dmft=1.8±0.02) (decayed-missing-filled- surfaces). The severity of the disease slightly increased along with its prevalence and reached 52% with dmft of 1.9 by 2004.[9-10] In 2005, Mohebbi and her associates reported that 3% to 33% of 1- to 3-year old children settled in Tehran experienced dental caries (mean dmft=1.1, CI=0.6-1.6).[11] These studies reported no significant relationship between level of education and occupation of parents with the rate of dmft.[10-11] Current epidemiological data available on dental caries status of Iranian children is mostly is based on “WHO Caries Assessment Criteria” (WHO-CAC) which records only the cavitated teeth.[5, 7, 12]

In 2002, the "International Caries Detection and Assessment System" (ICDAS) was first introduced by an international team of caries researchers.[13] It was later more developed as ICDAS-II in 2005.[14] The major purpose was to integrate several newly added criteria systems into one standard system for caries detection and assessment as well as to improve its consistency.[15] This system is considered as an evidence-based clinical caries scoring system and can be integrated to dental education and clinical practice. The index can improve the quality of diagnosis of dental caries. It is also applicable in research methodology and epidemiology. Moreover, it is a public health tool for community-based oral health promotion plans.[13, 16]

The ICDAS has been applied in a number of epidemiological settings as well as clinical practice and researches.[17-21] It is well used by European Global Oral Health Indicator Development Program to make the comparison of information easier between all of the union members.[22] As well as other countries, the ICDAS-II criteria have been applied in two different domains of two national investigations in Iran, clinical research and education.[23] Finally, a comprehensive article has also pointed out the risk factors of tooth decay in primary dentition.[24]

The present study discusses the community oral health implementation of ICDAS-II criteria in 2- to 3-year old Iranian children. This cross-sectional study presents caries experience of young children living in Tehran, the capital city of Iran. It also describes the pattern of dental caries development in this age group. Furthermore, it analyses the prevalence of this oral condition in relation to the ethnicity and the SES level of the participated children. 

## Materials and Method

**Sampling and sample size**

The samples were selected of children registered in public healthcare centers participated in the oral health promotion plan for different SES and ethnicity groups. This plan has the enrolment of nearly 85% of young children residing in Tehran.[25-27]According to the Center for Statistics of Iran, Tehran is the most hospitable city of Iran. Its population rates 9.5% of the whole country (8.2 out of 77.4 million), the 25^th^ crowded city of the world. It has the most immigrants’ settlement especially from the following provinces: Central Khorasan, Hamadan, East Azerbaijan, Kermanshah, Guilin, Luristan, Ardabil, Kurdistan, West Azerbaijan, and Northern Khorasan. Kermanshah and Kurdistan are correspondence to Kurdish; Ardabil, West and East Azerbaijan to Turkish; and Luristan to Lurs; while Hamadan and Northern Khorasan have both Turkish and Kurdish ethnic groups. Meanwhile, Afghans are the largest number of foreign immigrants entered in Iran.[28]

According to the ICDAS-II protocol, all tooth surfaces must be cleaned and freed of any dental plaque before oral examination.[29] The examination can be performed in either wet or dry conditions. Tooth surfaces are scored for both caries and restoration status leading to a two-digit number. The first digit represents the caries condition, while the second digit represents the restoration status. (Table 1)[7, 29] When using ICDAS method, air-drying can be substituted with damping with cotton wool/gauze. It should be mentioned that ICDAS codes can also be calculated as d_3_mf values.[7]

**Table 1:** ICDAS codes are described for caries severity, restoration status, and missing conditions.

**Caries severity**			**Restoration status**	
**Code**		**Description**	**Code**	**Description**
0		Sound tooth surface	0	Unrestored and unsealed
1		First visual change in enamel	1	Partial sealant- a sealant which does not cover all pits and fissures of the both surface
2		Distinct visual change in enamel	2	Full sealant
3		Localized enamel breakdown due to caries with no visible dentin	3	Tooth-colored restoration
4		Underlying dark shadow from dentin (with or without enamel breakdown)	4	Amalgam restoration
5		Distinct cavity with visible dentin	5	Stainless-steel crown
6		Extensive distinct cavity with visible dentin	6	Porcelain, gold or preformed metal crown or veneer
			7	Lost or broken restoration
			8	Temporary restoration
**Missing conditions**				
97	Missing due to caries			
98	Permanent tooth missing for other reasons			
99	Un-erupted tooth			

Stratified cluster random sampling was used as a proper method in this study. Participation in this investigation was fully voluntary. Districts of Tehran were stratified to three strata of North, Center, and South-regions. Then, three public health care centers were randomly selected in each stratum. Two hundred and thirty nine 24- to 36-month old children registered in public healthcare centers for the “Healthy Child Program” were recruited in this study. Being healthy with no systemic diseases, no milk-feeding with bottle or breast-feeding, and residency in Tehran were the inclusion criteria for participants. The use of any special medications by the children was the exclusion criteria.

According to PCA, participants of the study were categorized to five classes in which 0-20 represented the lowest, “1^st ^class”, 21-40 the next, “2^nd^ class”, 41-60, “3^rd^ class”, 61-80: “4^th ^class” and 81-100 the highest, “5^th^ class”. These classes were used in the following analysis of the study as “one factor” (SES).

**Calibration of the Examiners**

Two examiners were calibrated to screen child’s teeth using two methods. One examiner was trained for WHO method and the other for ICDAS-II method. The Kappa agreement was calculated between the first examiner and the second examiner, a faculty member of restorative and cosmetic dentistry department of the dental school. The second examiner was trained by a standard assessor in cariology, who was an approved expert in ICDAS-II coding system. The training module was a three-month electronic course. Twenty patients were examined in two rounds, ten patients in each round. Kappa coefficients analysis for validity and reliability of the first examiner was calculated. 

A total of 150 tooth surfaces were coded by the examiner and the responses were compared with the standard assessor. The first round of examination was carried out in pediatric dentistry clinic of school of dentistry. During eight work days (four work days per week) the examiner completed oral examination of 14 children with primary dentition. Tooth surfaces were coded according to ICDAS-II criteria and were photographed with a digital camera adjusted for manual setting. Later, images were sent to the benchmark examiner in San Antonio health research center via electronic mail for evaluation. Two consecutive rounds were performed using the standard training slides for ICDAS-II to determine the validity of coding. The final round (third) was carried out for the reliability. Agreement with the benchmark examiner was quantified by Kappa analysis. The scores less than 0.70 were considered to be acceptable as adequate agreement. 

**Oral Examination**

This cross-sectional study was carried out in public health care centers of Tehran during the course of 2 months. Mothers of selected children were interviewed by an educated and trained interviewer and were asked to complete a questionnaire for their demographic characteristics. All of the 239 recruited children were examined in the adjacent room in the knee-to-knee position by the calibrated examiners. All teeth were examined in wet and dry condition according to the proposed criteria for ICDAS-II index.[29]

First, tooth surfaces were cleaned with sterile gauze and flossed to eliminate debris and plaque, observed in wet condition under proper light of a head lamp. Then, teeth were examined for the second time after five second air-drying by means of a small rubber “camera clean dust ball” measured 12.5cm x 5.5cm with maximum pressure of 30 Kappa and minimum of 12 Kappa (HUIJIAQI; No.1869, China). If tactile was needed for confirmation of surface discontinuity, WHO ball-ended probe (CPITN) was used.[14] For WHO-CAC method, excess saliva was removed from the teeth and decayed teeth were coded as d_1_, d_2_, d_3_, and d_4_. The examiners recorded all codes using a digital voice recorder (Samsung; YV-120, Hong Kong) by its microphone fixed on their suites to reduce the potential errors during documentation. The oral examination followed a clock-wise sequence starting from the last tooth of the right upper quadrant (55 to 65 and then 75 to 85). All codes were entered into the Case Report Form (CRF) located in the last section of questionnaires.

**Ethical Considerations**

This study was approved by the Committee of Ethics on Research at Shahid Beheshti University of Medical Sciences. (No.614) The procedure and its schedule was clearly explained to the mothers. All recruited mothers accepted the procedure of this study through informed consent. All questions in the questionnaire were scored by specific codes. The data extracted from the completed questionnaires were entered into the software using the above codes. 

**Statistical Analysis**

The data was analyzed using SPSS (Version.21) and STATA (Version.11). All data obtained from interview and oral examination were entered into SPSS and double checked with regard to the original questionnaire and examination forms to eliminate the data entry errors. The corrected version was confirmed using frequency analysis. In addition, to get a specific “single factor” for SES, the data was entered into the STATA (Version.11), reconciled with the SPSS version, and double checked with original data by another research staff. The PCA assessed six variables (family monthly income, availability of computer at home, number of cars, level of father’s education, level of mother’s education, and municipal region of residency in Tehran) and emerged to “single factor” so called “SES”. These six economic indicators were previously described by Ghorbani *et al*.[30] as the socio-economic indicators for developing countries. The “SES” factor was described as a new variable for the SPSS software.

Descriptive statistics including frequencies, measures of center and spread were carried out to get the overall characteristics of samples and the prevalence. T-tests were used to compare mean values between subgroups. The one-way ANOVA and Tukey’s post hoc tests were used to compare the mean values of the frequency of ICDAS-II codes by SES and ethnicity. This analysis was also used to compare d_3,4_mft/d_3,4_mfs means between different ethnic and socio-economic groups.

Because of the presence of some common cultural characteristics between Kurdish, Lurs and Afghans, these ethnic groups were merged together and were classified as a three-level categorical predictor to implement regression analysis. In addition, by joining 1^st^ with 2^nd^ class as "low",3^rd^ as single class of "moderate", and 4^th^ with 5^th^ as "high", SES classes were redefined for effective analysis.

Binary logistic regression analysis was conducted to interpret the impact of three assumed predictors (gender, ethnicity, and SES) on the presence of dental caries among 2-to 3-year old children. This dichotomous dependent variable was described as caries-free (CF) and was allocated "zero" and "1" code to explain two conditions, absence or presence of caries, respectively. Multiple linear regression analysis was used to observe the impact of three categorical predictors on the frequency of ICDAS-II codes. 

## Results

In comparison with the standard examiners, Kappa coefficient analysis for validity of the first examiner was 0.7 for d_1_ and 1.0 for d_2-4 _and other components of dmfs. The intra-examiner reliability for WHO-CAC criteria was 1.0. Reliability and validity of the examiner for ICDAS-II was 0.97 and 0.72, respectively.

Among the 239 examined children with a mean age of 29.4±4.31 months, 47.7% (n=114) were male and 52.3% (n=125) were female. Most of the participants belonged to Persian (63.6%) ethnicity and the least were Afghan (1.3%) group. The detail of demographic characteristics of samples is explained in Ta ble 2. It shows that the distribution of the samples was nearly equal in different SES classes. In average, each child had 18 erupted teeth in his/her mouth (18±2). The mean value of caries-free surfaces (ICDAS-II code 00) among 239 children was 80.45±16.08. Overall, each child had approximately 80 caries-free surfaces in his/her mouth.

**Figure 1:**
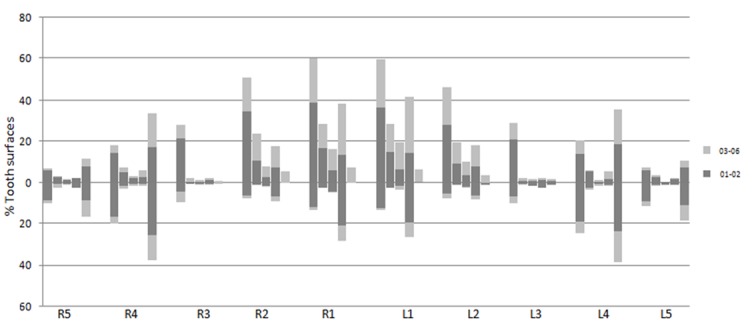
Oral examination of 24- to 36-month-old children demonstrates the pattern of caries development and frequency of different ICDAS-II codes among tooth surfaces. (Each bar corresponds to one surface of the tooth and named from the left to the right as buccal, labial, lingual, palatal, distal, mesial, occlusal, or incisal).

In addition, each participant experienced at least one gross cavitated tooth surface (ICDAS-II codes 05-06) before age three. (Figure 1)

In general, 10.87% of children were CF comprising of 11 males (9.6%), 15 females (12%). 61.1% of participants had cavitated caries (code 03-06) on their deciduous teeth until 36 months of age. A number 96 children (40.16%) had obvious dentin caries (codes 04-06), but had no filled surfaces. The mean of d_3,4_mft and d_3,4_mfs was 1.42±2.72 and 2.78±6.8, respectively. The percentage of children with d_3,4_mft=0 was 60.08% and SiC index=4.08. There was no significant difference between males and females in the mean of d_3,4_mft of 1.46±2.81 for males vs. 1.39±2.63 for females [*p*> 0.05, CI=(-0.63)-(0.76)] and d_3,4_mfs of 2.87±8.84 for males vs. 2.7±5.84 for females [*p*>0.05, CI=(-1.56)-(1.91)].

Labial surface of the upper right quadrant was the most common tooth surface affected by caries (Figure 1). The most observed code in this surface was 01 (51 out of 93 surfaces with code 01-02) for non-cavitated and 05 (26out of 51 surfaces with code 03-06) for cavitated lesions. Labial surfaces of the upper left quadrant followed the order.

Among 239 children, 7 teeth were missing (1.75% of the erupted teeth), 4 due to caries (code 97) and 3 due to trauma (code 98).

There was no significant difference between males and the females in tooth caries experience (Chi- Square=0.34, *p*= 0.56). Independent samples t-test showed a significantly higher frequency of code 01 in girls than in boys. (Table 2) The mean of the rest of ICDAS-II codes for dental caries was approximately equal between the two genders.

**Table 2:** Frequency of ICDAS-II codes among children based upon different gender, ethnicity, and socioeconomic (SES) classes.

**Variable**	**Subgroup**	**ICDAS-II codes (Mean±SD)**						
		**00**	**01**	**02**	**03**	**04**	**05**	**06**
**Gender**	Girls	79.04±16.44	4±5.13	3.36± 4.00	1.39± 2.33	0.17± 0.59	1.66± 3.35	1.12± 3.39
	Boys	81.99±15.6	2.75±3.78	3.59± 4.59	1.62±2.08	0.18± 0.5	1.72± 3.25	1.3± 5.78
	95%CI of mean difference	(-1.14)- (7.04)	(-2.4)- (-0.088)	(-0.86)- (1.32)	(-0.33)- (0.79)	(-0.12)- (0.15)	(-0.87)- (0.89)	(-1.01)- (1.37)
	P-value	0.785	0.023	0.101	0.632	0.819	0.877	0.477
**Ethnicity**	Persian	81.97±15.14	3.24±4.81	3.2± 4.37	1.34± 2.11	0.14± 0.47	1.17± 2.75	0.71± 2.6
	Turkish	79.21±16.85	3.78±4.29	4.1± 4.41	1.57± 2.18	0.19± 0.63	2.07± 3.26	1.73± 7.19
	Kurdish	66.2±15.49	3± 2.98	4.1± 2.99	3.5± 3.47	0.6± 0.84	7.8± 5.77	6.3± 7.05
	Lurs	88.14± 9.2	3.29±1.49	2.71±2.75	1.86± 1.67	0.29± 0.75	0.86± 0.9	0.00± 0.00
	Afghan	60.33± 26.5	5.33±8.38	2.33±4.04	0.67±1.15	0.00± 0.00	1± 1.73	0.33± 0.57
	P-value	0.003	0.869	0.613	0.046	0.122	0.000	0.004
**SES Class**	1^st^	79.08± 18.38	3.23±2.8	3.15± 4.25	1.52± 2.41	0.15± 0.46	1.85± 3.78	1.81± 8.04
	2^nd^	79.75± 16.08	4.04±4.41	3.4± 4.31	1.54± 2.2	0.23± 0.59	1.52± 3.49	0.79± 3.14
	3^rd^	73.88± 16.24	4.13±6.46	5.27± 4.68	2.1± 2.34	0.29± 0.77	2.33± 3.21	1.67± 4.03
	4^th^	82.69± 15.18	3.21± 5.32	3.21± 4.27	1.46± 2.43	0.19± 0.57	1.75± 3.55	1.35± 4.05
	5^th^	86.98 ±11.28	2.4 ± 2.54	2.3 ± 3.4	0.87 ± 1.26	0.02 ±0.14	0.98± 2.1	0.38 ±1.4
	P-value	0.002	0.34	0.013	0.116	0.171	0.373	0.536

One-way ANOVA analysis illustrated a significant difference between the mean of codes 00, 03, 05, and 06 among children of different ethnic groups. The Tukey’s range test also showed that these differences arose from significant differences between Persian and Kurdish ethnic groups. Kurdish children had the least code 00 and the most code 03-06 (Mean difference=15.81, SE=5.12, 95% CI= 1.72-29.9, *p*= 0.019). There was no significant difference between other ethnic groups.

ANOVA analysis also showed that the mean difference of the frequency of code 00 and code 02 was significant among the five socioeconomic classes. Further, Tukey’s post hoc analysis demonstrated that these differences were due to the significant difference between the moderate (3^rd^, p= 0.001, CI=4.29-21.92) and the highest (4^th^ and 5th, p= 0.006, CI=0.6-5.35) group of SES (Table 2).

One-way ANOVA demonstrated no considerable difference between various groups of SES classes in the mean of d_3,4_mft (*p*= 0.31) and d_3,4_mfs (*p*= 0.34). The analysis of variance declared a significant difference between various ethnic groups in the mean of d_3,4_mft (*p*=0.000) and d_3,4_mfs (*p*= 0.000). These differences were observed between Kurdish and other ethnic groups by the Tukey's test. The means of d_3,4_mft (6.9±5.02) and d_3,4_mfs (15.4±11.28) among Kurdish children showed considerable difference with other ethnic groups. (Table 3)

**Table 3:** The mean of d_3,4_mft/d_3,4_mfs are described among children from different ethnic groups.

**Ethnic groups**	** d_3,4_mft **				** d_3,4_mfs **			
	** M^a^±SD^b^**	** MD^c^**	** SE^d^**	** 95% CI^e^**	**M±SD**	**MD**	**SE**	**95% CI**
Persian	0.97±2.06	5.92	0.8	3.71-8.14	1.78±4.14	13.61	2.05	7.98-19.26
Turkish	1.72±2.87	5.18	0.83	2.88-7.49	3.46±9.1	11.93	2.13	6.08-17.79
Kurdish	6.9±5.02	Ref	Ref	Ref	15.4±11.28	Ref	Ref	Ref
Lurs	0.86±0.9	6.04	1.21	2.7-9.39	0.86±0.9	14.54	3.09	6.03-23.06
Afghans	1±1.73	5.9	1.62	1.43-10.37	1±1.73	4.13	4.13	3.03-25.7

a: Mean, b: Standard Deviation, c: Mean Difference, d: Standard Error, e: Confidence Interval

The Hosmer and Leme show test confirmed goodness of fit of the predicted probabilities of the model to those observed (*p*=1.000). Binary logistic regression analysis revealed that neither gender nor ethnicity and even SES had significant impact on prevalence of caries-free (CF) status of 2- to 3-year old children residing in Tehran, Iran. (Table 4)

**Table 4:** Factors related to the presence of dental caries among 2- to 3-year old children in Tehran as explained by binary logistic regression analysis.

**Parameters**	**S.E**	**OR**	**%95 CI**	**P-value**
Gender Female Male	Ref 0.427	Ref 0.853	Ref 0.36-1.97	Ref 0.71
Ethnicity Persian Turkish Kordish & Lurs & Afghan	1.072 1.136 Ref	2.61 1.56 Ref	0.31-21.37 0.16-14.47 Ref	0.37 0.69 0.46
SES Low Moderate High	0.506 0.541 Ref	0.584 1.03 Ref	0.21-1.57 0.35-2.99 Ref	0.28 0.94 0.507

Multiple linear regression analysis showed the significant impact of ethnicity on the frequency of codes 05 and 06, as well. Gender also represented a considerable impact upon the frequency of ICDAS-II code of 01. (Table 5)

**Table 5:** Factors related to the frequency of ICDAS-II codes among 2- to 3-year-old children in Tehran as explained by multiple linear regression analysis.

**Parameters**	**ICDAS-II codes**	**S.E**	**B**	**%95 CI**	**P-value**
Gender	00 01 02 03 04 05 06	2.05 0.59 0.56 0.28 0.072 0.41 0.604	-3.5 1.31 -0.17 -0.18 -0.005 0.04 -0.08	(-7.54)-(0.53) (0.15)-(2.47) (-1.28)-(0.92) (-0.75)-(0.38) (-0.14)-(0.13) (-0.77)-(0.86) (-1.27)-(1.10)	0.08 0.027^*^ 0.75 0.52 0.94 0.90 0.89
Ethnicity	00 01 02 03 04 05 06	1.64 0.47 0.44 0.22 0.05 0.33 0.48	-3.23 0.18 0.306 0.403 0.094 1.36 1.15	(-6.64)-(-0.07) (-.74)-(1.11) (-0.57)-(1.18) (-0.04)-(0.85) (-0.19-(0.2) (0.71)-(2.02) (0.2)-(2.12)	0.05 0.69 0.49 0.08 0.1 0.000^*^ 0.01^*^
SES	00 01 02 03 04 05 06	1.18 0.34 0.32 0.16 0.04 0.24 0.34	2.24 -0.43 -0.19 -0.10 -0.02 0.08 -0.001	(-0.88)-(4.56) (-1.09)-(0.23) (-.812)-(0.44) (-0.42)-(0.22) (-0.10)-(0.05) (-0.36)-(0.55) (-0.68)-(0.68)	0.59 0.2 0.55 0.54 0.56 0.71 0.99

^* ^Significant difference

## Discussion

This epidemiological study was a cross-sectional investigation using ICDAS-II criteria to detect dental caries among young children, a comparable method with previous studies. It was also designed to assess the correlation of some assumed risk factors.[30] In comparison with the two previous national surveys, the percent age of caries free (CF) children living in Tehran diminished from the mean national value of 53% in 1999[9] to 48% in 2004[10] to 10.87% in recent years. In 2005, the CF rate was reported 67% by Mohebbi’s cross- sectional study for the children living in Tehran.[11] These differences may attribute to the diversity of measurement methods along with the duration of study. The previous studies that used WHO-CAC method of dmft,[12] which considered only dentinal caries (d_3_) as the decay “d” component of the dmft.

The 40.16% (n=96) of children with codes 04-06 (dentinal caries) along with the 61.08% of children with d_3,4_mft=0 in the present study can be compared with the results of the previous studies. The present result can be regarded to the higher rate of immigration from other cities into the capital city of Tehran and similar rates of move-out during the period of 2006 to 2012.[28] Thus, dental caries and the mean d_3,4_mft of 1.42 vs. 1.2 of Mohebbi’s study shows an increasing trend of the CF rate during recent years among young children in Tehran.

As one of the few studies carried out among similar age group of children using similar method of diagnosis (ICDAS-II), Cadavid *et al*.[17] conducted an investigation among 2.5- to 4-year old children in Medellin, Colombia. The total prevalence of 89% of non-cavitated and cavitated dental caries among the 2- to 3-year old children living in Tehran was comparable with their study which concluded that 25% of children had no sign of caries on their tooth surfaces. Another Colombian study performed on 3- to 5-year old children of Cartagena City in 2010 reported prevalence of 88.9% (51.7% cavitated lesions considering the cut-off of 03 and 37.5% non-cavitated lesions) caries experience.[20] A recent study also stated a prevalence of 69.9% caries experience among 3-year-old children living in Medellin, Colombia.[19]

Regarding the pattern of caries development among recruited children in the present study, the upper right quadrant of the child’s mouth was the most opportunistic site to start and progress dental caries. The direction of the dental caries progress was from midline including interproximal surfaces of the central incisor teeth to the posterior sites of the child’s mouth. The dental caries developed both in maxillary and mandibular teeth and involved labial and occlusal surfaces. It follows the progressive pattern as described by ADA for early childhood caries (ECC) detection.[31-32]

## Conclusion

Irrespective of the gender, ethnicity and SES, there is a high distribution of dental caries among young children which merits a priority attention for community oral health promotion. Building public health policies to promote oral health of children from birth is recommended. Impact of ethnicity as a predictor of the frequency of dentinal caries confirms that minorities are much more susceptible to the EEC condition. Implementing community healthcare programs for pregnant mothers and continuing primary care of infants after delivery is an invaluable service.[33]
